# Solid Organ Transplant Graft-Versus-Host Disease in a Kidney/Pancreas Transplant Patient: Use of Chimerism Testing and a Rare Presentation of Cutaneous GVHD

**DOI:** 10.1155/2022/6539808

**Published:** 2022-03-09

**Authors:** Amrita Goyal, Jeremy Allred, Raja Kandaswamy, Erik B. Finger, Daniel O. Keys, Samy Riad, Alessio Giubellino, Daniel D. Miller, Christine G. Lian, Shernan G. Holtan

**Affiliations:** ^1^Department of Dermatology, University of Minnesota, Minneapolis, MN, USA; ^2^Division of Hematology, Oncology, and Transplantation, Department of Medicine, University of Minnesota, Minneapolis, MN, USA; ^3^Department of Surgery, University of Minnesota, Minneapolis, MN, USA; ^4^Division of Nephrology and Hypertension, Department of Medicine, University of Minnesota, Minneapolis, MN, USA; ^5^Department of Pathology, University of Minnesota, Minneapolis, MN, USA; ^6^Division of Dermatopathology, Department of Pathology, Brigham and Women's Hospital, Minneapolis, MN, USA

## Abstract

*Introduction*. Solid organ transplant graft-versus-host disease (SOT-GVHD) is a rare phenomenon in which recipients of solid organ transplant develop GVHD due to the presence of donor lymphocytes in the graft. SOT-GVHD most often occurs in patients receiving small bowel or liver transplants. Diagnosis is typically via identification of lymphocytic infiltration on histopathology and molecular demonstration of donor T cell chimerism in the target organ. The gastrointestinal (GI) system is the most common target of SOT-GVHD, and one estimate places long-term survival of patients with SOT-GVHD at 20% at 5 years. In this report, we present the case of a patient with sequential kidney and pancreas transplant who developed SOT-GVHD targeting host lymphocytes, skin, and liver, with a long period of stability before treatment with antithymocyte globulin. Peripheral blood chimerism testing was used to track response to therapy. Remarkably, he survived 1.5 years despite recurrent infections before dying of unrelated causes.

## 1. Clinical Case

A man, aged approximately 50 years, with a history of type 1 diabetes mellitus (T1DM) received a deceased-donor kidney transplant for end-stage renal disease. Two years later, he received a deceased-donor pancreas transplant for endocrine pancreatic insufficiency (time *T*). Prior to the pancreas transplant, the patient was markedly anemic, although his white blood cell (WBC) count, absolute neutrophil count (ANC), and platelets were in the normal range. His immunosuppression regimen included tacrolimus, mycophenolate mofetil, and prednisone.

Two months after the pancreas transplant, the patient presented with transaminitis, marked leukopenia, and a pruritic rash across the chest and extremities. Liver biopsy demonstrated scattered hepatocyte necrosis and bile duct damage with focal ductopenia, consistent with drug toxicity versus graft-versus-host disease (GVHD). T cell subsets on flow cytometry showed persistent severe absolute T lymphopenia. Bone marrow biopsy showed slightly hypocellular marrow (20-30%) with trilineage hematopoiesis, slightly atypical megakaryocytes, and 1% blasts with no evidence for leukemia or lymphoma. Peripheral blood showed anemia and leukopenia. Peripheral blood chimerism studies were performed using fluorescently labelled oligonucleotide primers for 16 highly polymorphic genetic markers, and results of peripheral blood were compared to previously analyzed tissue from the donor and recipient. This revealed 96% donor (pancreas) T cells and 100% recipient myeloid cells in the peripheral blood. Donor T cell chimerism was also detected in his liver. Together, all of these data were consistent with SOT-GVHD targeting multiple organs. Although the transaminitis improved without additional treatment, he continued to have persistent immunodeficiency (absolute lymphocyte count of 0 on CBC) and subsequently developed severe CMV and HSV esophagitis.

**Figure 1:**
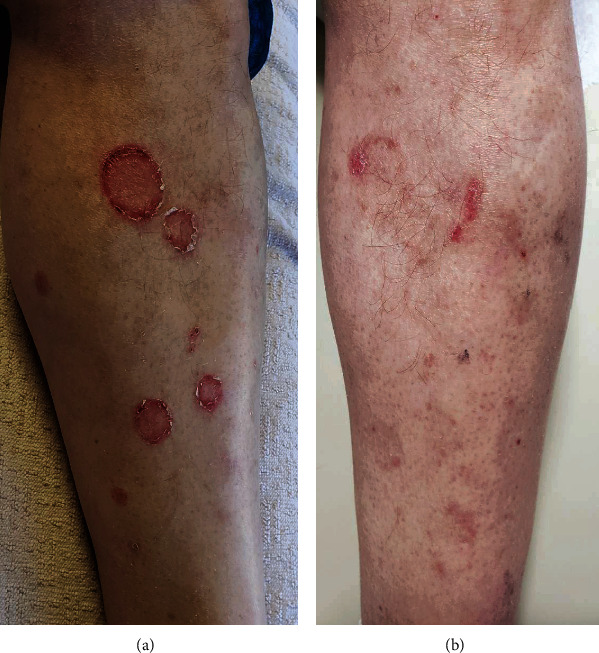
Solid organ transplant-associated cutaneous graft-versus-host disease (a) prior to antithymocyte globulin (ATG) and (b) 3 months after ATG.

**Figure 2:**
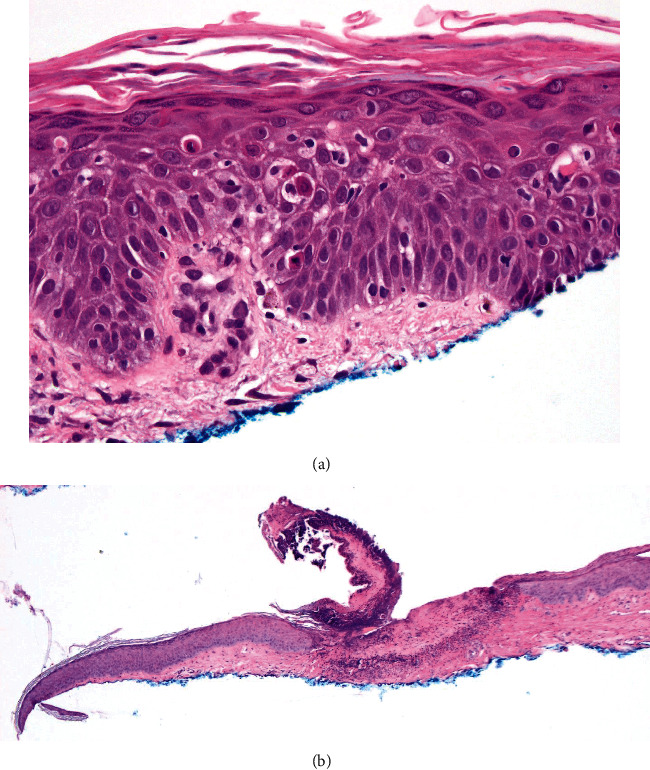
(a) Necrotic keratinocytes and satellitosis on a background of interface change and spongiosis suggestive of graft versus host disease in a patient with pancreas transplant. (b) Erosion composing the border of what clinically appeared to be a coronoid lamella but on microscopy is scale crust and necrotic epidermis.

A year after his pancreas transplant, due to persistent lymphopenia and risk of infection, the patient underwent treatment with antithymocyte globulin (ATG, 15 mg/kg daily for 7 days, with 100 mg methylprednisolone daily, *T* + 12 months); no additional changes were made to his immune suppression regimen at that time. He was found at that time to have a group of 2-6 cm annular plaques with eroded raised border and central clearing on the left shin. Although clinically these were concerning for eruptive porokeratosis or tinea (Figure 1(a)), biopsy revealed necrotic keratinocytes with satellitosis and foci of interface vacuolar change on a background of spongiosis, consistent with mild GVHD (Figure 2). The biopsy also demonstrated a significant erosion, which reflected what clinically appeared to be the border of the porokeratosis-like lesion. Within 4 weeks of treatment with ATG, these lesions had resolved with scarring (Figure 1(b)).

Two months after ATG, there was improvement of ALC to 0.2 and hemoglobin to 11.3, as well as normalization of liver function tests. Blood chimerism analysis at that time showed CD3+ cells to be 13% donor (pancreas) and 87% recipient; 100% myeloid cells were of recipient origin (see Table 1). This was accompanied by an improvement in the hemoglobin (see Table 2). Retesting of clonality at 4 months post-ATG demonstrated 100% recipient lymphocyte chimerism.

Unfortunately, 17 months after the pancreatic transplant, he died due to procedure-related complications unrelated to his SOT-GVHD.

## 2. Discussion

SOT-GVHD is a rare, poorly recognized, often rapidly fatal consequence of solid organ transplants including the pancreas and small intestine. Pancreas-related SOT-GVHD has been reported in only a handful of cases and is typically lethal [1–3]. There are several aspects of this case that are unique: (1) the targeting of the multiple organs by donor T cells with spontaneous clinical remission in the skin and liver, (2) the utilization of peripheral blood chimerism studies to track disease progress, and (3) the patient's reversion to complete host T cell chimerism post-ATG.

Because of its rarity, literature regarding the treatment of SOT-GVHD is limited. Therapeutic options are limited and typically focus on immune modulation including cessation of immune suppression, ATG, or high-dose steroids [4]. In the case of this patient with SOT-GVHD causing life-threatening lymphopenia and recurring skin wounds, treatment was chosen primarily to target the circulating alloreactive donor T cells which were thought to be the primary drivers of the patient's disease. Eradication of donor T cells would allow for recovery of host T cells and reduction of infection risk.

Measurement of peripheral blood chimerism was the key to this patient's diagnosis and treatment. This is one of the most important laboratory tests in the field of bone marrow transplantation (BMT). It allows identification of host versus donor cell fraction and assessment of the degree of engraftment in BMT. There have been other cases of the use of chimerism to detect SOT-GVHD; however, few have used this technology to track response to therapy in this setting. This case demonstrates another important application of chimerism testing, determining the degree of engraftment in SOT-GVHD and tracking response to therapy.

It is unusual that the GVHD targeted only the lymphoid lineage, leaving the myeloid lineage intact, as evidenced by his generally near-normal hemoglobin and platelets. When SOT-GVHD targets the marrow compartment, the usual result is complete marrow aplasia resulting in pancytopenia.

Another interesting facet of this case is the presentation of porokeratosis-like GVHD; however, pathology revealed that these lesions had an eroded border without coronoid lamella and were hence not porokeratosis. This highlights the importance of biopsy in patients with SOT-GVHD given significant disease pleomorphism. This diagnosis is strengthened by the fact that as his chimerism studies improved after treatment with ATG, these lesions disappeared.

Overall, numerous facets of this case are highly instructive, including the use of chimerism studies to track GVHD improvement in a SOT patient and a rare presentation of cutaneous GVHD.

## Figures and Tables

**Table 1:** Chimerism data before and after ATG administration.

		Postpancreas transplant, prior to ATG (*T* + 10)		Postpancreas transplant, two months after ATG (*T* + 14 months)	Postpancreas transplant, four months after ATG (*T* + 16 months)
Lymphoid (CD3+)	Pancreas transplant (*T*)	3% recipient 96% donor (pancreas)	ATG (*T* + 12 months)	87% recipient 13% donor (pancreas)	100% recipient
Myeloid (CD33/66b+)		100% recipient		100% recipient	100% recipient

**Table 2:** Lab values before and after pancreas transplant and ATG administration.

	Prior to pancreas transplant	Two months after pancreas transplant (*T* + 2 months)	At the time of ATG treatment (*T* + 12 months)	Two months after ATG treatment (*T* + 14 months)	Four months after ATG (*T* + 16 months)	Reference range
WBC (×10^9^ cells/L)	6.0	8.5	1.9	2.0	1.8	4.0 − 11.0 × 10^9^ cells/L
ALC (×10^9^ cells/L)	0.7	0.0	0.0	0.2	0.1	0.7 − 5.3 × 10^9^ cells/L
ANC (×10^9^ cells/L)	4.4	5.3	1.3	1.1	1.3	1.6 − 8.3 × 10^9^ cells/L
Hemoglobin (g/dL)	7.5	8.0	9.2	11.3	11.6	13.3 − 17.7 g/dL
Platelets (×10^9^ cells/L)	159	308	248	272	325	150 − 450 × 10^9^ cells/L

ATG: antithymocyte globulin; WBC: white blood cell count; ALC: absolute lymphocyte count; ANC: absolute neutrophil count; g: grams; L: liter; dL: deciliter.

## Data Availability

Data is available upon request.
